# Identification of therapeutic targets for neonatal respiratory distress: A systematic druggable genome-wide Mendelian randomization

**DOI:** 10.1097/MD.0000000000042411

**Published:** 2025-05-16

**Authors:** Hang Yuan, Feng Gao, HongLi Wang

**Affiliations:** a Department of Neonatal Intensive Care Center (NICU), Henan Provincial Key Medicine Laboratory of Nursing, Henan Provincial People’s Hospital, People’s Hospital of Zhengzhou University, Zhengzhou, Henan, China.

**Keywords:** cis-eQTL, colocalization, Mendelian randomization, neonatal respiratory distress

## Abstract

Currently, there remains a significant gap in effective pharmacologic interventions for neonatal respiratory distress syndrome (NRDS). To address this critical unmet medical need, we aimed to systematically identify novel therapeutic targets and preventive strategies through comprehensive integration and analysis of multiple publicly accessible datasets. In this study, we employed an integrative approach combining druggable genome data, cis-expression quantitative trait loci (cis-eQTL) from human blood and lung tissues, and genome-wide association study summary statistics for neonatal respiratory distress. We performed two-sample Mendelian randomization (TSMR) analysis to investigate potential causal relationships between druggable genes and neonatal respiratory distress. To strengthen causal inference, we performed Bayesian co-localization analyses. Furthermore, we conducted phenome-wide Mendelian randomization (Phe-MR) to systematically evaluate potential side effects and alternative therapeutic indications associated with the identified candidate drug targets. Finally, we interrogated existing drug databases to identify actionable pharmacological agents targeting the identified genes. All 3 genes (LTBR, NAAA, CSNK1G2) were analyzed by Bayesian co-localization (PH4 > 75%). CSNK1G2 (lung eQTL, odds ratio [OR]: 0.419, 95% CI: 0.185–0.948, *P* = .037; blood eQTL, OR: 4.255, 95% CI: 1.346–13.455, *P* = .014; Gtex whole blood eQTL, OR: 4.966, 95% CI: 1.104–22.332, *P* = .037). LTBR (lung eQTL, OR: 0.550, 95% CI: 0.354–0.856, *P* = .008; blood eQTL, OR: 0.347, 95% CI: 0.179–0.671, *P* = .002; Gtex whole blood eQTL, OR: 0.059, 95% CI: 0.0.007–0.478, *P* = .008). NAAA (lung eQTL, OR: 0.717, 95% CI: 0.555–0.925, *P* = .011; Gtex whole blood eQTL, OR: 0.660, 95% CI: 0.476–0.913, *P* = .012). Drug repurposing analyses support the possibility that etanercept and asciminib hydrochloride may treat neonatal respiratory distress by activating LTBR. This study demonstrated that LTBR, NAAA, and CSNK1G2 may serve as promising biomarkers and therapeutic targets for NRDS.

## 1. Introduction

Neonatal respiratory distress syndrome (NRDS) is characterized by progressive dyspnea and respiratory failure in newborns shortly after birth, primarily attributed to insufficient alveolar surfactant production.^[1,2]^ This condition can lead to severe complications including respiratory failure, cardiac dysfunction, and life-threatening infections.^[3,4]^ While alveolar surfactant replacement therapy has been established as the primary preventive and therapeutic approach for NRDS, its clinical efficacy remains limited.^[5,6]^ Furthermore, there is a notable paucity of research investigating potential preventive or therapeutic targets for NRDS. Consequently, elucidating the underlying pathogenesis of this condition and identifying novel therapeutic targets represent crucial areas of research interest.

In recent years, the rapid advancement of biotechnology has significantly propelled the development of multi-omics research, an interdisciplinary field that integrates large-scale data from diverse biological domains, including transcriptomics and metabolomics. This integrative approach has provided robust evidence and sophisticated tools for identifying potential disease biomarkers and therapeutic targets.^[7]^ Among various research methodologies, Genome-Wide Association Studies (GWAS) have emerged as a powerful approach for investigating genetic factors underlying complex diseases. By conducting genome-wide typing of high-density genetic markers (e.g., SNPs) across large-scale population DNA samples, GWAS enables comprehensive identification of genes associated with disease onset, progression, and therapeutic response.^[8]^ Furthermore, the integration of expression quantitative trait loci (eQTL) analysis with GWAS datasets has proven particularly valuable in elucidating genetic variations that influence gene expression patterns related to disease phenotypes.^[9]^ Notably, cis variants, which are located on the same chromosome and in close proximity to the transcribed gene unit, have demonstrated greater utility than trans variants in identifying functionally relevant phenotypic changes. This characteristic makes cis variants particularly valuable for exploring the genetic mechanisms underlying gene expression regulation.^[10,11]^

There has been a surge in reports highlighting the successful development of novel therapeutic agents, with the identification of disease-specific drug targets serving as a critical prerequisite. To streamline the exploration of potential drug targets, researchers have introduced the concept of “druggable genomes,” which has significantly accelerated the discovery of new therapeutic targets.^[12,13]^ As an integral component of genomics research, the druggable genome comprises genes encoding proteins with inherent potential to serve as drug targets, primarily due to their capacity to bind therapeutic compounds effectively. The application of druggable genome analysis has markedly enhanced both the efficiency and success rate of novel drug target identification.^[14,15]^ Furthermore, Mendelian randomization (MR), an innovative methodological approach that utilizes genetic variants strongly associated with exposure factors as instrumental variables (IVs) to assess causal relationships between exposures and outcomes, has provided robust evidence in causal inference studies.^[16]^ The integration of druggable genome analysis with cis-expression quantitative trait loci (cis-eQTL) MR has emerged as a powerful research strategy, successfully identifying potential therapeutic targets for various complex diseases, including amyotrophic lateral sclerosis, idiopathic pulmonary fibrosis, and type 2 diabetes mellitus.^[17–19]^

In this study, we systematically integrated druggable genomic data, transcriptomic profiles, and GWAS data of NRDS to identify potential preventive and therapeutic targets for this condition.

## 2. Methods

**Figure 1.**
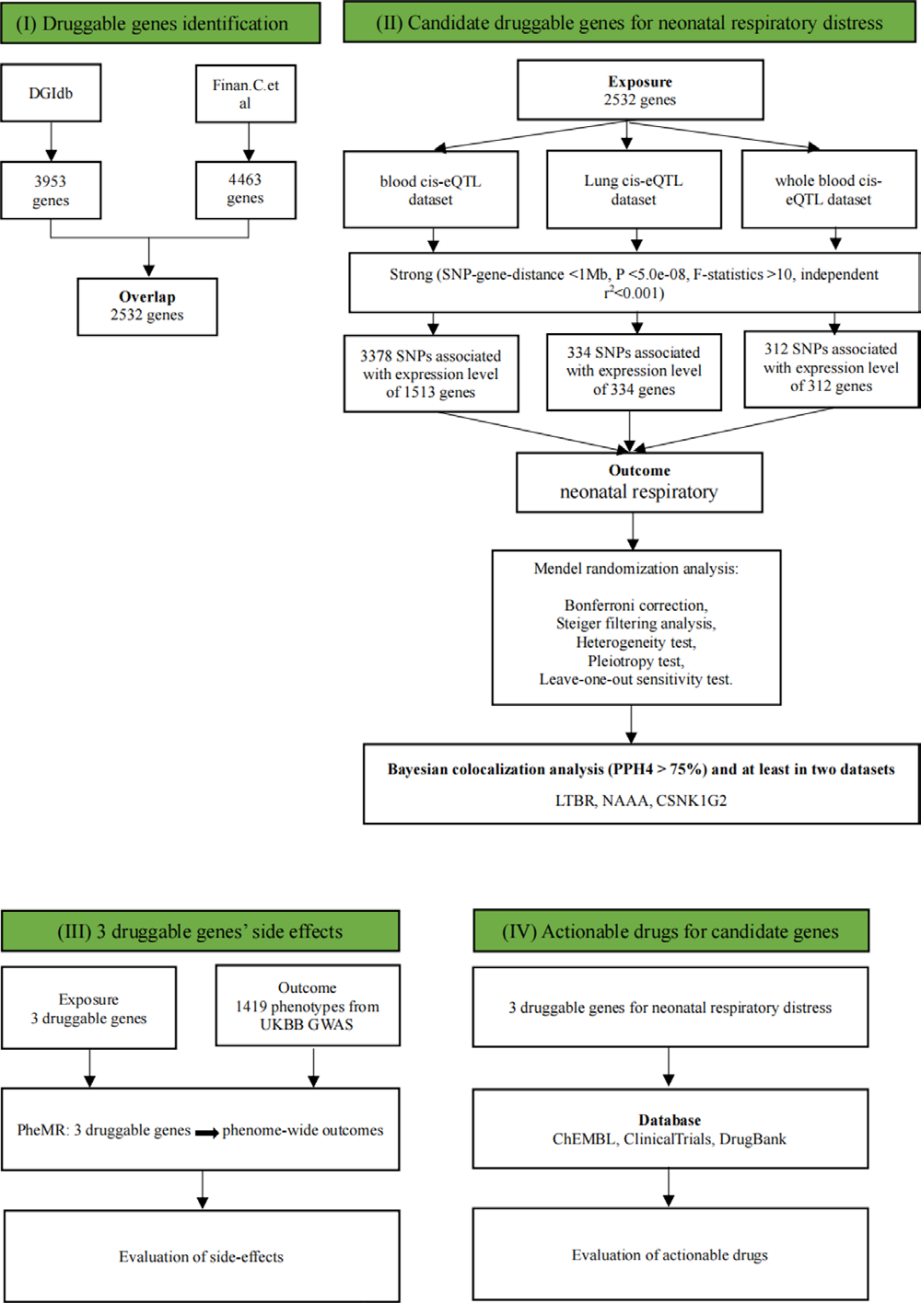
We curated a comprehensive set of 2532 known druggable genes from the DGIdb database, following the methodology established by Finan et al to construct eQTL tools for these druggable genes, we integrated eQTL data derived from human blood (eQTLGen), whole blood (GTEx), and lung tissues (GTEx). Through this process, we identified independent genetic variants significantly associated with druggable gene expression, which served as instrumental variables (IVs). These IVs were specifically located within 1 Mb upstream (cis) and downstream of the coding sequence. In our MR analysis, these IVs were employed to identify potential causal genetic variants associated with neonatal respiratory distress. Furthermore, we implemented a Bayesian co-localization approach to detect shared causal genetic variants across traits. To evaluate the clinical relevance of our findings, we conducted phenome-wide MR (Phe-MR) analyses using UK Biobank GWAS data to assess potential side effects and additional therapeutic indications of the prioritized druggable genes. Finally, we systematically investigated the availability and clinical development status of these genes through comprehensive database searches and website evaluations. eQTL = expression quantitative trait loci, eQTLGen consortium = expression quantitative trait locus generating consortium, DGIdb = drug-gene interaction database, GTEx = genotype-tissue expression project, GWAS = genome-wide association study, IV = instrumental variable, MR = Mendelian randomization, Phe-MR = whole-phenomenon Mendelian randomization analysis.

The schematic representation of the research framework is illustrated in Figure 1. The analysis was conducted using aggregated datasets, with all necessary informed consent and ethical approvals having been secured in the original studies from which the data were derived.

## 3. Data sources

### 3.1. druggable genes

We retrieved druggable genes from 2 primary sources: the drug-gene interaction database (DGIdb v5.0.8, https://www.dgidb.org/downloads)^[20]^ and and the seminal work of Finan et al.^[13]^ DGIdb represents a comprehensive repository specializing in drug-gene interactions, integrating diverse data sources encompassing drug mechanisms of action, gene mutations, clinical trial outcomes, and other relevant information. This database serves as an essential resource for researchers and healthcare professionals, facilitating advancements in personalized medicine and drug discovery. Specifically, we accessed the February 2022 release of DGIdb, which contains curated drug-gene interaction data mapped to Entrez gene identifiers.^[20]^ Furthermore, Finan et al groundbreaking study established critical connections between GWAS-identified loci associated with complex diseases and druggable genes, thereby providing a robust framework for drug target identification and validation.^[13]^ The integration of these 2 complementary data sources significantly enhances the reliability and comprehensiveness of our analysis.

## 4. eQTL dataset

We obtained blood cis-eQTL data encompassing 19,250 gene transcripts from 31,684 individuals through the eQTLGen consortium.^[21]^ Furthermore, we acquired comprehensive cis-eQTL data from both lung tissue and whole blood samples through the Genotype-Tissue Expression (GTEx) Consortium, comprising a cohort of 715 individuals (Table 1).

**Table 1:** Data sources for the MR analysis in the current study.

Type of dataset	Data subtype	Source	Sample size	Population	Download site
Druggable genome	DGIdb	Freshour.SL, et al 2020			https://www.dgidb.org/downloads
	Prior druggable gene	Finan.C et al 2017			Finan C, et al PMID: 28356508.
QTL datasets	Lung cis‐eQTL	GTEx Consortium 2020 Science	715	European	https://gtexportal.org/home/datasets.
	Whole blood cis‐eQTL	GTEx Consortium 2020 Science	715	European	https://gtexportal.org/home/datasets.
	Blood cis‐eQTL	eQTLGen Consortium (Võsa U, et al 2021)	31 684	European	https://www.eqtlgen.org/cis-eqtls.html
GWAS summary	Respiratory distress of newborn	FinnGen	Cases: 171, Controls: 218,527.	European	https://www.finngen.fi/en GWAS ID (finn-b-P16_RESPI_DISTR_NEWBO)
	1419 Phenotypes	UK Biobank (Zhou, et al 2018)	408 961	European	https://www.leelabsg.org/resources

cis‐eQTL = cis-expression quantitative trait loci, DGIdb = drug-gene interaction database, eQTLGen = expression quantitative trait locus generating consortium, GWAS = genome-wide association study, GTEx = genotype-tissue expression.

## 5. GWAS datasets for neonatal respiratory distress

We retrieved genome-wide association study (GWAS) summary statistics for NRDS from the FinnGen database, comprising 171 cases and 218,527 controls (Table 1).

## 6. Instrument selection

Mendelian randomization methods employ single nucleotide polymorphisms (SNPs) that exhibit strong associations with the exposure of interest as IVs to evaluate the causal relationship between exposure and outcomes. These IVs must satisfy 3 fundamental assumptions: the IV demonstrates a direct association with the exposure; the IV remains independent of potential confounding factors; and the IVs are independent of the outcome variable.^[22]^ To ensure the robustness of our analysis while minimizing potential pleiotropic effects and maintaining the integrity of the 3 core MR assumptions (specifically, that IVs influence outcomes exclusively through their effect on exposure without alternative pathways), we implemented stringent selection criteria. These included applying a genome-wide significance threshold (*P* < 5 × 10^−8^) and requiring an *F*-statistic ≥ 10 to identify strong IVs.^[23]^

## 7. Two-sample Mendelian randomization

Following the identification of candidate druggable gene IV, we extracted corresponding variants from the GWAS dataset for neonatal respiratory distress. To evaluate the exposure-outcome association, we employed both the Wald ratio and inverse variance weighting methods. The *P*-values of the available genes were corrected by Bonferroni (Bonferroni threshold: Lung eQTL at 0.05/334, blood eQTL at 0.05/1531, and whole blood eQTL at 0.05/312). Comprehensive sensitivity analyses were conducted using MR-Egger, weighted mode, and weighted median approaches, complemented by multidirectionality assessment through MR-Egger intercept analysis.^[24]^ Potential outliers were identified using the MR-PRESSO global test, with significant SNP outliers being systematically excluded from subsequent analyses.^[25]^ Statistical significance thresholds were set at *P* < .05 for both heterogeneity and multidirectionality analyses. To establish causal directionality, we implemented Steiger analysis using the “TwoSampleMR” R software package, effectively mitigating potential reverse causality bias. The causal direction was determined as follows: true (*P* < .05) for exposure-to-outcome effects, false (*P* < .05) for reversed effects, and inconclusive (*P* ≥ .05).^[26]^ All statistical analyses were performed using R software (version 4.1.2) with specialized packages including TwoSampleMR, MR-PRESSO, ensuring methodological rigor and reproducibility.

## 8. Bayesian colocalization

Bayesian colocalization is a robust statistical approach designed to assess whether the observed association signals between 2 traits originate from the same underlying genetic variants. This methodology evaluates 5 distinct hypotheses through posterior probability calculations: PPH0, indicating no association between the traits; PPH1, where only trait 1 shows genetic association; PPH2, where only trait 2 demonstrates genetic association; PPH3, where both traits exhibit genetic associations but through distinct variants; and PPH4, where both traits share the same causal genetic variant. In our analysis, we considered potentially druggable genes and neonatal respiratory distress to share the same genetic variant when the posterior probability of PPH4 exceeded 75%.^[27]^ The Bayesian co-localization analysis was conducted using the “coloc” package (version 5.1.0) in R (https://cran.r-project.org/web/packages/coloc/), following standard implementation protocols.

## 9. Phenome‐wide MR analysis

To comprehensively investigate the causal relationships between the identified druggable genes and various disease traits, we conducted a phenome-wide MR (Phe-MR) analysis. This systematic approach aimed to maximize the identification of potential causal associations and provide robust evidence to facilitate novel drug development. In this study, we utilized GWAS data comprising 1419 phenotypes derived from 408,961 White British participants of European ancestry in the UK Biobank. The phenotypic data were processed using the scalable and accurate implementation of generalized mixed models (SAIGE) method, as previously described by Zhou et al, which enabled the analysis of more than 1400 binary phenotypic samples. The GWAS summary statistics were obtained from the SAIGE database (https://www.leelabsg.org) to ensure statistical reliability, with detailed phenotypic information presented in Table 1.

## 10. Actionable drugs for candidate genes

We conducted a comprehensive assessment of the identified druggable genes for potential drug development by leveraging 3 major biomedical databases: DrugBank (https://go.drugbank.com), ChEMBL (https://www.ebi.ac.uk/chembl), and ClinicalTrials.gov (https://www.clinicaltrials.gov).

## 11. Results

### 11.1. Druggable genome

To identify potential drug targets, we initially extracted 3953 potentially druggable genes from the DGIdb database (Table S1, Supplemental Digital Content, https://links.lww.com/MD/O869) and obtained an additional 4463 druggable genes from the comprehensive study conducted by Finan et al (Table S2, Supplemental Digital Content, https://links.lww.com/MD/O869). To enhance the reliability and validity of our target selection, we performed a rigorous comparative analysis of these 2 datasets. This analysis resulted in the identification of 2532 high-confidence druggable genes that were consistently validated by both sources and had been officially annotated by the Human Genome Nomenclature Committee (Table S3, Supplemental Digital Content, https://links.lww.com/MD/O869).

## 12. Candidate druggable genes for neonatal respiratory distress

**Figure 2.**
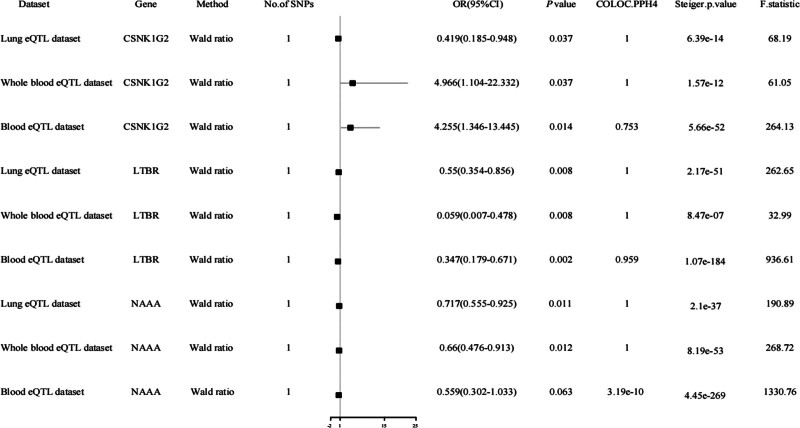
Summary of druggable genes for neonatal respiratory distress identified by MR and Bayesian co-localization analysis. In order to make IV more effective and to reduce pleiotropy at the same time, this study mainly used single SNPs as exposure tools. A Steiger *P*-value < .05 is considered true, which indicates that IV causes outcome. Figure 1 shows that the 3 druggable genes passed Mendelian randomization and Bayesian co-localization analysis. The eQTL dataset and neonatal respiratory distress were considered to have the same variant if the posterior probability of PPH4 was > 75%. The strength of the association between exposure and outcome is showed by OR. OR is defined as: if OR > 1, exposure may promote the outcome; if OR < 1, exposure may suppress the outcome. OR = odds ratio.

We overlapped 2532 potentially druggable genes with genes in the human blood, whole blood, and lung eQTL datasets, and then extracted genetic variation within a 1 Mb range on either side of the coding sequences of the overlapping druggable genes. We obtained 334 SNPs associated with 334 druggable genes in the human lung tissue cis-eQTL, 3378 SNPs associated with 1531 druggable genes in the human blood cis-eQTL, and 312 SNPs associated with 312 druggable genes in the human whole blood cis-eQTL as representative of the IVs exposed to MR analysis (Fig. 1). Next, we performed MR analysis of exposure using the GWAS Abstract dataset for neonatal respiratory distress. And after correction, we found that 3 druggable genes simultaneously present in at least 2 eQTL databases (Bonferroni corrected *P* < .05) were causally associated with neonatal respiratory distress (Figs. 1 and 2).

To identify potential drug targets sharing genetic variants with NRDS, we employed Bayesian co-localization analysis to determine whether the causal signals identified in our MR study originated from identical genetic variants. Our findings revealed 3 promising druggable genes (LTBR, NAAA, CSNK1G2) that demonstrated consistent co-localization across multiple quantitative trait locus (QTL) datasets. Notably, while the NAAA gene failed to show co-localization in the blood expression quantitative trait loci (eQTL) dataset, it exhibited shared genetic variants with NRDS in both lung and whole blood eQTL datasets (Fig. 2 and Table S4, Supplemental Digital Content, https://links.lww.com/MD/O869). The analysis of transcript levels revealed tissue-specific associations with NRDS risk. In lung tissue, each standard deviation (SD) increase in CSNK1G2 expression was associated with a reduced risk of NRDS (odds ratio [OR]: 0.419, 95% CI: 0.185–0.948, *P* = .037). Conversely, elevated CSNK1G2 transcript levels in blood samples from eQTLGen (OR: 4.255, 95% CI: 1.346–13.455, *P* = .014) and whole blood samples from GTEx (OR: 4.966, 95% CI: 1.104–22.332, *P* = .037) were associated with increased NRDS risk, highlighting the complex tissue-specific regulatory mechanisms underlying this condition.

The analysis of transcript levels from multiple databases revealed significant associations with neonatal respiratory distress. In the eQTLGen Blood database, each 1 SD increase in LTBR transcript levels was associated with a reduced risk of neonatal respiratory distress (OR: 0.347, 95% CI: 0.179–0.671, *P* = .002). Similar protective associations were observed in GTEx databases, with LTBR transcript levels showing reduced risk in both lung tissue (OR: 0.550, 95% CI: 0.354–0.856, *P* = .008) and whole blood (OR: 0.059, 95% CI: 0.007–0.478, *P* = .008). For NAAA transcript levels, a 1 SD increase was associated with lower neonatal respiratory distress in lung tissue (OR: 0.717, 95% CI: 0.555–0.925, *P* = .011) and GTEx whole blood (OR: 0.660, 95% CI: 0.476–0.913, *P* = .012). However, NAAA transcript levels in blood from the eQTLGen database did not reach statistical significance (OR: 0.559, 95% CI: 0.302–1.033, *P* = .063). Importantly, our findings passed the Steiger filter test, providing robust support for our hypothesis that the IVs directly influence the outcome variable (Fig. 2 and Table S4, Supplemental Digital Content, https://links.lww.com/MD/O869). These results collectively suggest a protective role of LTBR and NAAA transcript levels against neonatal respiratory distress.

## 13. Phe‐MR analysis of neonatal respiratory distress‐associated candidate druggable genes

We conducted PheWAS-MR analyses on 1149 diseases and traits from the UK Biobank, using IVs consistent with previously identified druggable genes associated with neonatal respiratory distress (6 SNPs in 3 genes, excluding those with nonsignificant Bayesian colocalization). Causal effects were considered statistically significant at *P* ≤ 1 × 10⁻^4^. Increased LTBR transcript levels were associated with a reduced risk of urinary incontinence (OR: 1.074, 95% CI: 1.036–1.112, *P* = 8.9 × 10⁻⁵). However, no associations were found between CSNK1G2 and NAAA and any of the 1419 diseases, suggesting that drugs targeting these genes may not have significant adverse effects (Table S4, Supplemental Digital Content, https://links.lww.com/MD/O869 and Fig S1, Supplemental Digital Content, https://links.lww.com/MD/O870).

## 14. Actionable drugs for target genes

We have genetically evaluated 3 drug candidates for neonatal respiratory distress to assess their preclinical and clinical developmental potential (Table 2). While agents targeting CSNK1G2 have been specifically investigated, compounds related to the other 2 genes have been evaluated in clinical trials for other indications but have not been explored for neonatal respiratory distress treatment. Notably, Etanercept^[28]^ and Asciminib hydrochloride,^[29]^ which function as LTBR gene activators and maintain normal LTBR gene expression, emerge as promising therapeutic candidates for neonatal respiratory distress through their LTBR activation mechanism. In contrast, Nabiximols^[30]^ and Cannabidiol,^[31]^ which act as NAAA inhibitors, appear less suitable due to their potentially adverse effects on neonatal respiratory distress.

**Table 2:** Finding actionable drugs for target genes.

Gene	Molecule type	Compounds	Action type	Clinical development activities	Gene molecular function
NAAA	Small Molecule	Nabiximols	Inhibitor	Investigational. treatment of neuropathic pain from multiple sclerosis and for cancer pain.	DNA-binding transcription factor binding/hydrolase activity.
	Small Molecule	Cannabidiol	Inhibitor	Approved/investigational. treatment of Lennox-Gastaut syndrome and Dravet syndrome.	
CSNK1G2					ATP binding/histone H2AS1 kinase activity/protein serine kinase activity
LTBR	Small Molecule	Etanercept	Agonist	Approved/ investigational. Treatment of severe rheumatoid arthritis and moderate to severe plaque psoriasis	Cytokine activity/signaling receptor binding/tumor necrosis factor receptor binding
	Small Molecule	Asciminib hydrochloride	Agonist	Approved/investigational. Treatment of CGL	

CGL = chronic granulocytic leukemia, LTBR = lymphotoxin beta receptor.

## 15. Discussion

Based on the comprehensive datasets analyzed in this study, we have identified robust evidence supporting the genetic co-localization of 3 promising drug target genes (LTBR, NAAA, and CSNK1G2). Through systematic evaluation of their clinical development potential, we demonstrate that Etanercept and Asciminib hydrochloride may represent effective therapeutic agents for NRDS through their activation of LTBR. Furthermore, our findings suggest that the expression profiles of these 3 genes could potentially serve as valuable biomarkers for the prevention and management of neonatal respiratory distress.

The lymphotoxin beta receptor gene (LTBR ) encodes a member of the tumor necrosis factor receptor superfamily, known as the lymphotoxin beta receptor. This receptor plays a pivotal role in immune system regulation, particularly in the development and maintenance of lymphoid tissues and intercellular signaling pathways, primarily through its interaction with lymphotoxin beta.^[32]^ Functionally, LTBR mediates critical cellular processes including apoptosis and proliferation, thereby contributing significantly to both adaptive and innate immune responses.^[33,34]^ Notably, recent studies have revealed an additional role of LTBR in regulating the expression of leucine and tryptophan biosynthetic genes in Corynebacterium glutamicum, an important amino acid-producing bacterium.^[35]^ Our MR study indicates that elevated LTBR gene transcription levels may confer protection against NRDS. While the precise mechanistic link between LTBR and NRDS remains to be fully elucidated, existing evidence suggests that both maternal antenatal systemic inflammatory indices^[36]^ and neonatal airway inflammatory manifestations^[37]^ are significant contributors to NRDS development. These findings suggest that modulation of inflammatory responses and enhancement of immune system functionality may represent promising therapeutic strategies for NRDS management. The primary pharmacological activators of LTBR currently identified are Etanercept and Asciminib hydrochloride. However, our MR analyses were unable to establish optimal therapeutic parameters, including dosage regimens and treatment durations, for these agents. These critical clinical considerations warrant further investigation through well-designed prospective studies and randomized controlled trials to establish evidence-based therapeutic protocols.

The CSNK1G2 gene has been identified as one of the key genes demonstrating common genetic co-localization associated with NRDS. Notably, increased transcript levels of CSNK1G1 have been established as a significant risk factor for NRDS. CSNK1G2 encodes a protein that belongs to the protein tyrosine kinase family, specifically within the cell cycle protein-dependent kinase 1 family, which is crucial for various biological processes. Recent studies have revealed a novel function of CSNK1G2 in regulating oxidative stress response and modulating reactive oxygen species levels.^[38]^ Additionally, CSNK1G2 has been shown to play a pivotal role in sensitizing breast cancer cells to tamoxifen (TAM) toxicity by differentially regulating the PI3K/AKT/mTOR/S6K and ERK signaling pathways.^[39]^ Despite these advancements, the association between CSNK1G2 and neonatal respiratory distress remains unexplored. Furthermore, the development of effective CSNK1G2 inhibitors for clinical applications remains an unmet need, highlighting a critical gap in current research and therapeutic strategies.

N-Acetylethanolamine acid hydrolase (NAAA) is predominantly localized within the endochromosome-lysosome compartment of both innate and adaptive immune cells. NAAA-targeted pharmacological agents demonstrate significant therapeutic potential in the management of human inflammatory diseases.^[40]^ Emerging research has revealed that N-acyl-ethanolamine amidase (NAAA) plays a crucial role in mitigating intestinal fibrosis through the modulation of macrophage activity.^[41]^ Furthermore, empirical evidence indicates that NAAA expression is markedly elevated in rat alveolar macrophages.^[42]^ Our investigation has identified a correlation between upregulated NAAA transcript levels and decreased susceptibility to neonatal respiratory distress. While NAAA-related compounds, such as Nabiximols and Cannabidiol, have received clinical approval, these agents primarily function as NAAA inhibitors. Consequently, there is a compelling need to explore and develop NAAA agonists as potential therapeutic interventions for neonatal respiratory distress.

This study demonstrates several notable strengths that enhance its scientific value and practical implications. Primarily, our approach incorporated comprehensive druggable gene databases for initial screening, ensuring that the identified candidate genes were directly associated with drug targets. This strategic selection significantly improves the potential success rate of future drug development while reinforcing the reliability of our findings. Secondly, our methodology integrated cis-acting variants of gene expression with GWAS data on neonatal respiratory distress, followed by rigorous Bayesian co-localization analysis. The cross-validation of results using independent datasets from different sources further strengthens the accuracy and credibility of our analytical outcomes. Thirdly, through systematic exploration of online databases, we successfully identified clinical drug information corresponding to candidate pharmacogenetic genes, providing valuable insights into their potential therapeutic applications for neonatal respiratory distress.

However, certain limitations should be acknowledged. First, the scarcity of comprehensive eQTL and pQTL data specific to neonatal blood in public databases presents a significant constraint, potentially limiting our ability to fully elucidate the pathogenesis of neonatal respiratory distress. Second, the exclusive reliance on GWAS data from European populations in this study may affect the generalizability of our findings. Inclusion of diverse ethnic groups in future research would enhance the robustness and applicability of the results across different populations.

## 16. Conclusion

Our findings unveil promising therapeutic targets for NRDS, with 3 potential druggable genes (LTBR, NAAA, and CSNK1G2) emerging as candidates for further investigation. However, the translational potential of these targets derived from MR analyses requires rigorous validation through well-designed clinical trials. Future studies should systematically evaluate both the therapeutic efficacy and safety profiles of these molecular targets, while considering the inherent limitations of MR-based approaches in clinical translation.

## Acknowledgments

We are sincerely grateful to the researchers who made the GWAS data publicly available.

## Author contributions

**Conceptualization:** Hang Yuan.

**Data curation:** Hang Yuan.

**Formal analysis:** Hang Yuan.

**Funding acquisition:** Hang Yuan.

**Investigation:** Hang Yuan.

**Methodology:** Hang Yuan.

**Project administration:** Hang Yuan.

**Resources:** Hang Yuan.

**Software:** Hang Yuan.

**Validation:** Hang Yuan.

**Visualization:** Hang Yuan.

**Writing – original draft:** Hang Yuan.

**Writing – review & editing:** Hang Yuan, Feng Gao, HongLi Wang.

## Supplementary Material
