# Supplementary material for: Identification of therapeutic targets for neonatal respiratory distress: A systematic druggable genome-wide Mendelian randomization
Source: Medicine (Baltimore). 2025 May 16;104(20):e42411. doi: 10.1097/MD.0000000000042411 (PMC12091608; doi:10.1097/MD.0000000000042411)

Figure.S1. Manhattan plot of Phe-MR analysis for druggable genes.

A-B. Manhattan plot of Phe-MR analysis for CSNK1G2.

C-D. Manhattan plot of Phe-MR analysis for LTBR.

E-F. Manhattan plot of Phe-MR analysis for NAAA.

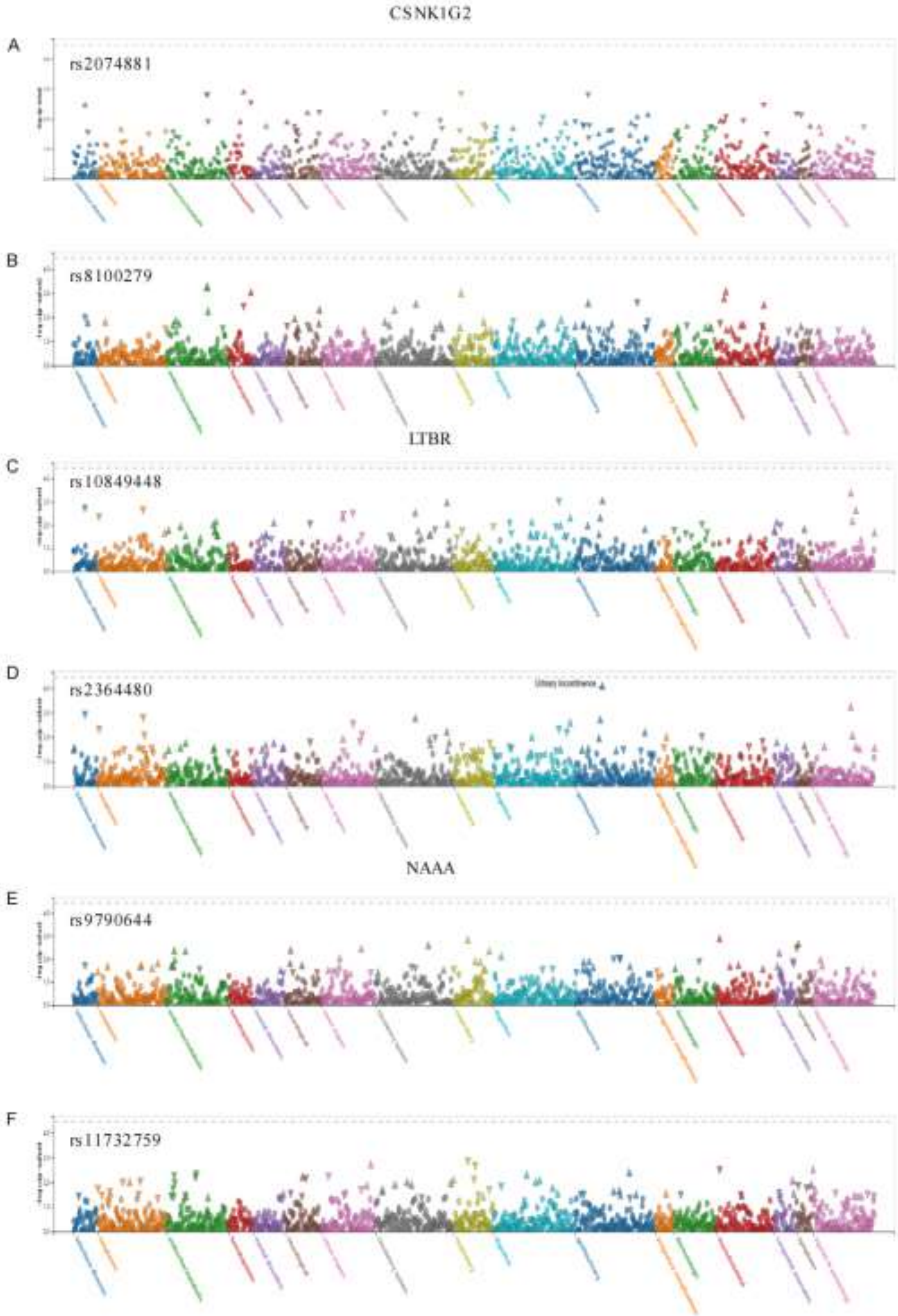

Supplement: Supplementary file 2 [file medi-104-e42411-s002.pdf]
